# Factors associated with deep infiltrating endometriosis versus ovarian endometrioma in China: a subgroup analysis from the FEELING study

**DOI:** 10.1186/s12905-018-0697-7

**Published:** 2018-12-22

**Authors:** Yi Dai, Yingfang Zhou, Xinmei Zhang, Min Xue, Pengran Sun, Jinhua Leng, Charles Chapron

**Affiliations:** 1 Department of Gynecology and Obstetrics, Peking Union Medical College Hospital, Chinese Academy of Medical Science and Peking Union Medical College, Beijing, China; 2 Department of Gynecology and Obstetrics, Peking University First Hospital, Beijing, China; 3 Department of Gynecology and Obstetrics, Women’s Hospital School of Medicine Zhejiang University, Hangzhou, China; 4 Department of Gynecology and Obstetrics, The Third Xiangya Hospital of Central South University, Changsha, China; 5 Medical Department, Ipsen (Beijing) Pharmaceutical Science and Technology Development Co., Ltd, Beijing, China; 6 Department of Gynecology and Obstetrics II and Reproductive Medicine, Centre Hospitalier Universitaire Cochin, Baˆtiment Port Royal, 53 avenue de l’Observatoire, Paris, 75014 France

**Keywords:** Ovarian endometrioma (OMA), Deep infiltrating endometriosis (DIE), FEELING study, Chinese women

## Abstract

**Background:**

To compare potential factors associated with deep infiltrating endometriosis (DIE) versus ovarian endometrioma (OMA) among endometriosis patients in China.

**Methods:**

A subgroup analysis of factors associated with DIE versus OMA was performed in Chinese women from the FEELING study. This study included 156 OMA patients and 78 DIE patients. Retrospective information on symptoms and previous medical history was collected via face-to-face interviews; patients also completed a questionnaire to provide information on current habits. Univariate and multivariate logistic regression analyses were conducted to identify potential factors that are associated with DIE versus OMA.

**Results:**

From univariate analysis, women who were married, at older age, had any siblings, prior pregnancy, or longer time since age at menarche on the day of visit were more likely to be diagnosed with DIE (*P* < 0.05). Also, the incidence of previous uterine surgery, menstrual and ovulatory disorders, deep dyspareunia, and gastrointestinal symptoms during menstruation were major factors that were significantly associated with the diagnosis of DIE (*P* < 0.05). Multivariate analysis showed that women with any siblings, gastrointestinal symptoms during menstruation, or eating a greater number of fruit/vegetables per day were more likely to be diagnosed with DIE. Meanwhile, eating organic food and experiencing stress were major factors that are associated with the diagnosis of OMA.

**Conclusions:**

The findings provide additional information on the potential risk factors that are associated with DIE, compared with OMA among Chinese endometriosis patients. The results may help to better understand DIE versus OMA, and aid in earlier risk stratification and diagnosis of the patients.

**Trial registration:**

NCT01351051. Registered 10 May 2011.

## Background

Endometriosis, known as ectopic growth of endometrium, is an enigmatic disease that has been consistently associated with dysmenorrhoea, deep dyspareunia, cyclical premenstrual symptoms, and even infertility [1–7]. It affected 10.8 million people worldwide according to the Global Burden of Disease Study in 2015 [8]. The estimates of the prevalence of endometriosis range from 2 to 10% of women of reproductive age [9], to 50% of infertile women [10]. Hormonal therapy, surgery or their combinations are the main therapeutic options available to treat endometriosis. However, surgical treatment is difficult and can be risky for the most severe forms of the disease, especially in cases of DIE [11, 12]. Endometriosis imposes a substantial economic burden on society due to the necessity of costly medical and surgical treatments, and the indirect costs associated with a reduced quality of life and lost work productivity [13, 14]. Various genetic, epigenetic, environmental, or dietary factors are implicated in the aetiology of the disease [15–19]. However, despite the advances in our understanding of endometriosis, the pathogenesis of the disease is still unclear.

Macroscopically, endometriosis falls into three distinct entities: superficial peritoneal endometriosis (SUP), cystic ovarian endometriosis or endometrioma (OMA), and deeply infiltrating endometriosis (DIE) [20]. OMA and DIE are the two most important manifestations of endometriosis, and the study by Holt and Weiss even suggested only consideration of OMA and DIE as the “definite disease”. “Definite disease” was defined as laparoscopically visible ovarian endometriomas of any size, endometriotic pelvic implants deeper than 5 mm, or any visible ectopic endometrial implants in the presence of ovarian or pelvic adhesions without another explanation [21]. In addition to heterogeneity in locations, these two forms of endometriosis present a kaleidoscopic variation in size, color, and depth of invasion [22, 23], which may in part contribute to the vast variation in symptomatology and severity of the diseases. Clinically, OMA is reportedly the most common [24], accounting for 17–44% of patients with endometriosis [25]. Despite the lower prevalence, however, DIE is consistently known to be strongly associated with severe pain, poor quality of life and sexual dysfunction [26, 27]. It has long been established as the most aggressive form of the disease [28]. However, the heterogeneity of the disease and the absence of definite clinical symptoms may cause long delays in diagnosis (3–12 years) and/or mis-diagnosis of the disease. Identification of the potential risk factors that are associated with DIE or OMA may provide further information on early diagnosis of the disease. Definite diagnosis of the disease at an early stage may help to better management of the symptoms, which in turn may reduce disease progression and prevent subsequent infertility in patients [29]. Moreover, it may also be helpful to better understand the pathogenesis of the disease, thus to develop curative and even preventive treatments that are not available now.

OMA and DIE have long been postulated as two separate disease entities, and thus may have different pathogenesis and pathophysiology [30]. There is a clinical need to identify and develop methods and/or tools to facilitate the early risk stratification of patients with OMA and DIE. However, few studies have attempted to elucidate the differences between OMA and DIE in Chinese women. To investigate the potential factors that may help the clinical diagnosis of DIE versus OMA in China, the present study was carried out using a set of wide-ranging clinical, lifestyle, and environmental factors from the previous multi-country, case-control study (Factors associated with the development of Endometrioma and dEep infiLtratING endometriosis, FEELING, NCT 01351051) [8].

## Methods

### Patients

This was a subgroup analysis of Chinese women from the FEELING study that was conducted at 4 hospital gynecologic departments in China (Peking Union Medical College Hospital, Beijing; Peking University First Hospital, Beijing; Women’s Hospital School of Medicine Zhejiang University, Hangzhou, Zhejiang; Third Xiangya Hospital of Central South University, Changsha, Hunan). A total of 546 Chinese women were recruited, of whom 390 were histologically diagnosed with endometriosis and 156 were non-endometriosis. Among the patients with endometriosis, 156 women were classified as SUP, 156 were OMA and the remaining 78 were in the DIE group. The study protocol was approved by the institutional review boards at each participating institution in China. Patients provided written informed consent to allow their medical data to be collected, analyzed, and shared with regulatory authorities.

### Inclusion & exclusion criteria

Patients meeting the following criteria were included in the study. Inclusion criteria were 1) histologically diagnosed with OMA and/or DIE; 2) aged between 18 and 41 years; 3) had undergone laparoscopy or laparotomy for a benign gynaecological indication within the preceding 3 months; 4) DIE was defined as endometriotic lesions infiltrating deeper than 5 mm beneath the peritoneum surface [8]. 5) DIE was ranked as the most severe endometriotic lesion, followed by OMA [31]. Patients with concomitant DIE and OMA were included into the DIE group (from the 78 patients with DIE, 33 (43.2%) also had OMA,). The exclusion criteria were 1) women in pregnancy; 2) with malignancy or mental disease; 3) uncooperative with the study design.

### Study design

All patients were surveyed at their usual appointments with the physicians. Data on baseline socio-demographic characteristics, gynaecological and medical history were collected by the investigators using an electronic Case Report Form. A face-to-face interview was conducted to obtain retrospective data on symptoms and previous medical history (endometriosis history and status, pre-surgical complaints, surgery details). The patients were also required to complete a questionnaire on their health-related behaviors during the post-surgical visit, which included dietary habits (consumption of alcohol, coffee, convenience food, sugar, meat/fish, milk, black tea, vegetables, fat, salt), health status (overall health status, exercise), mood (stress, depression, anxiousness, sleep disorders) and environment (pollution exposure, smoking environment, sun exposure, employment status). All data were collected retrospectively and no intervention was applied in this study.

### Statistical analysis

All statistical analyses were performed using SAS® software, version 9.1 (SAS Institute Inc., Cary, NC). Factors associated with DIE versus OMA were investigated using logistic regression analysis model. Univariate logistic regression analysis was performed to screen variables for association with DIE versus OMA. Variables with a *P* value below the 20% significance level in the univariate analysis were entered into the multivariate analysis. Variables including previous surgical diagnosis, hormonal treatment for endometriosis, and infertility were also added in a multivariate logistic regression model. A stepwise elimination analysis was performed using a significance level of 10% to enter the model and a significance level of 5% to retain them. Significant associations were determined at *P* < 0.05 level.

## Results

Data from 78 DIE and 156 OMA Chinese patients were obtained from the FEELING study. The demographic and baseline characteristics of the Chinese patients were summarized in Table 1. The following factors were identified to be significantly associated with the development of DIE/OMA on the basis of the univariate logistic regression analysis. Women with older age (OR = 4.32 95% CI: 2.29–8.17, *P* < 0.001), having any siblings (OR = 2.91 95% CI: 1.45–5.85, *P* = 0.003) or prior pregnancy (OR = 1.96, 95% CI: 1.08–3.58, *P* = 0.028) were more likely to be diagnosed with DIE. By contrast, those who were single were more likely to be diagnosed with OMA when compared with the married ones (OR = 0.18, 95% CI: 0.05–0.62, *P* = 0.042). Other characteristics including body mass index (BMI), educational level, blood type, co-morbidities (including cancer, allergic disease and other diseases), having been breastfed, and a family history of endometriosis were not significantly associated with the diagnosis of DIE (Table 1).

**Table 1:** Demographic and baseline characteristics of the women with DIE vs OMA

Factors	OMA (*n* = 156)	DIE (*n* = 78)	OR (95% CI)	*P*
Mean age (years)	31.17 ± 4.97	34.47 ± 4.54	4.32 [2.29;8.17]	< 0.001
BMI (kg/m^2^)				0.354
> = 18.5 to < 22	81 (51.9%)	33 (42.3%)	ref	
< 18.5	28 (17.9%)	13 (16.7%)	1.14 [0.53;2.47]	
> =22 to < 25	34 (21.8%)	21 (26.9%)	1.52 [0.77;2.99]	
> =25	13 (8.3%)	11 (14.1%)	2.08 [0.85;5.10]	
Educational level				0.205
University or business school	100 (64.1%)	45 (57.7%)	ref	
Primary school + High school	24 (15.4%)	21 (26.9%)	1.94 [0.98;3.85]	
Vocational or professional school	18 (11.5%)	7 (9.0%)	0.86 [0.34;2.22]	
Polytechnic or equivalent	14 (9.0%)	5 (6.4%)	0.79 [0.27;2.34]	
Blood type				0.483
A	57 (36.8%)	28 (37.3%)	ref	
B + AB	50 (32.3%)	29 (38.7%)	1.18 [0.62;2.25]	
O	48 (31.0%)	18 (24.0%)	0.76 [0.38;1.55]	
Rhesus				0.988
+	153 (98.7%)	75 (100.0%)	ref	
-	2 (1.3%)	0 (0.0%)	< 0.01 [NE]	
Marital status				0.042
Married	122 (78.2%)	74 (94.9%)	ref	
Single	27 (17.3%)	3 (3.8%)	0.18 [0.05;0.62]	
Free union	5 (3.2%)	1 (1.3%)	0.33 [0.04;2.88]	
Divorced/separated/Widowed	2 (1.3%)	0 (0.0%)	< 0.01 [NE]	
Co-morbidities ^a^				0.532
No	149 (95.5%)	73 (93.6%)	ref	
Yes	7 (4.5%)	5 (6.4%)	1.46 [0.45;4.75]	
Prior pregnancy				0.028
No	63 (40.4%)	20 (25.6%)	ref	
Yes	93 (59.6%)	58 (74.4%)	1.96 [1.08;3.58]	
Having been Breastfed				0.086
No	142 (91.0%)	76 (97.4%)	ref	
Yes	14 (9.0%)	2 (2.6%)	0.27 [0.06;1.21]	
Having any siblings				0.003
No	54 (34.6%)	12 (15.4%)	ref	
Yes	102 (65.4%)	66 (84.6%)	2.91 [1.45;5.85]	
Family history of endometriosis				0.477
No	149 (95.5%)	76 (97.4%)	ref	
Yes	7 (4.5%)	2 (2.6%)	0.56 [0.11;2.76]	

^a^Includes cancer, allergic disease and other diseases

In view of menstrual characteristics and pre-surgical complaints, women who had a longer time since age at menarche on the day of visit were more often associated with DIE (OR = 3.92, 95% CI: 2.12–7.26, *P* < 0.001). The incidence of previous uterine surgery (OR = 2.10, 95% CI: 1.13–3.89, *P* = 0.018), menstrual and ovulatory disorders (OR = 3.13 95% CI: 1.14–8.57, *P* = 0.026), deep dyspareunia (OR = 3.37, 95% CI: 1.74–6.56, *P* < 0.001), and gastrointestinal symptoms during menstruation (OR = 1.79, 95% CI: 1.02–3.15, *P* = 0.042) were significant factors associated with DIE (*P* < 0.05, Table 2). However, incidence of non - cyclic chronic pelvic pain, dysmenorrhoea, pain at time of ovulation, infertility, urinary symptoms during menstruation, practice of vaginal douching, previously surgical diagnosis, hormonal treatment for endometriosis, and use of combined oral contraceptive pills were not significantly associated with DIE (*P* > 0.05, Table 2).

**Table 2:** Menstrual and pre-surgical complaints of the women with DIE vs OMA

Factors	OMA (*n* = 156)	DIE (*n* = 78)	OR (95% CI)	*P*
Time since age at menarche (years)^a^	18.09 ± 5.10	21.31 ± 4.52	3.92 [2.12;7.26]	< 0.001
Lifelong menstrual cycles				0.799
Always + Generally regular	151 (96.8%)	75 (96.2%)	ref	
Irregular	5 (3.2%)	3 (3.8%)	1.21 [0.28;5.19]	
Non-cyclic chronic pelvic pain				0.203
No	129 (82.7%)	59 (75.6%)	ref	
Yes	27 (17.3%)	19 (24.4%)	1.54 [0.79;2.99]	
Menstrual/ovulatory disorders^b^				0.026
No	149 (95.5%)	68 (87.2%)	ref	
Yes	7 (4.5%)	10 (12.8%)	3.13 [1.14;8.57]	
Dysmenorrhoea				0.410
No	46 (29.5%)	15 (19.2%)	ref	
Yes	110 (71.5%)	63 (81.8)		
Pain at time of ovulation				0.467
No	144 (92.3%)	74 (94.9%)	ref	
Yes	12 (7.7%)	4 (5.1%)	0.65 [0.20;2.08]	
Deep dyspareunia				< 0.001
No	135 (87.1%)	52 (66.7%)	ref	
Yes	20 (12.9%)	26 (33.3%)	3.37 [1.74;6.56]	
Infertility				0.168
No	121 (77.6%)	54 (69.2%)	ref	
Yes	35 (22.4%)	24 (30.8%)	1.54 [0.83;2.83]	
Gastrointestinal symptoms				0.042
No	109 (69.9%)	44 (56.4%)	ref	
Yes	47 (30.1%)	34 (43.6%)	1.79 [1.02;3.15]	
Urinary symptoms				1.000
No	140 (89.7%)	70 (89.7%)	ref	
Yes	16 (10.3%)	8 (10.3%)	1.00 [0.41;2.45]	
Previous surgical diagnosis				0.338
No	1a31 (84.5%)	62 (79.5%)	ref	
Yes	24 (15.5%)	16 (20.5%)	1.41 [0.70;2.84]	
Previous uterine surgery				0.018
No	126 (80.8%)	52 (66.7%)	ref	
Yes	30 (19.2%)	26 (33.3%)	2.10 [1.13;3.89]	
Hormonal treatment				0.058
No	122 (78.2%)	52 (66.7%)	ref	
Yes	34 (21.8%)	26 (33.3%)	1.79 [0.98;3.29]	
Combined oral contraceptive pills				0.484
No	154 (98.7%)	76 (97.4%)	ref	
Yes (Ex-user + Current user)	2 (1.3%)	2 (2.6%)	2.03 [0.28;14.66]	

^a^On the visit day, ^b^Menstrual/ovulatory disorders (ovulatory disorders, amenorrhea, menorrhagia)

When it comes to health-related behaviours, women eating a greater number of fruit or vegetables per day were more likely to be diagnosed with DIE (OR = 2.15, 95% CI: 1.01–4.57, *P* = 0.047). However, women eating organic food were significantly associated with the diagnosis of OMA (Rarely + Sometimes: OR = 0.25 95% CI: 0.10–0.63; Often + Always: OR = 0.22, 95% CI: 0.07–0.72, *P* = 0.010). Eating a greater number of fried food portions per week (OR = 0.03, 95% CI: 0.00–0.49, *P* = 0.013), using thermal facilities (saunas, spas etc) (OR = 0.44, 95% CI: 0.22–0.87, *P* = 0.018) and suffering from stress (OR = 0.30, 95% CI: 0.12–0.77, *P* = 0.012) were significant factors that associated with OMA (Table 3).

**Table 3:** Health-related behaviors of the women with DIE vs OMA

Factors	OMA (*n* = 156)	DIE (*n* = 78)	OR (95% CI)	*P*
Eating organic food				0.010
Never	8 (5.2%)	14 (18.2%)	ref	
Rarely + Sometimes	126 (81.3%)	55 (71.4%)	0.25 [0.10;0.63]	
Often + Always	21 (13.5%)	8 (10.4%)	0.22 [0.07;0.72]	
No. of fruit/vegetables per day	2.45 ± 3.13	3.53 ± 4.52	2.15 [1.01;4.57]	0.047
No. of fried food per week	1.59 ± 1.99	0.98 ± 0.97	0.03 [0.00;0.49]	0.013
Sleep				
6–8 h per night	103 (66.5%)	53 (67.9%)	ref	0.969
More than 8 h per night	38 (24.5%)	18 (23.1%)	0.92 [0.48;1.77]	
Less than 6 h per night	14 (9.0%)	7 (9.1%)	0.97 [0.37;2.55]	
Sun exposition (> 1 h) days/year	147.28 ± 125.03	125.07 ± 119.16	0.99 [0.96;1.01]	0.203
Use thermal facilities				0.018
No	107 (68.6%)	65 (83.3%)	ref	
Yes	49 (31.4%)	13 (16.7%)	0.44 [0.22;0.87]	
Stress level				
Not stress at all	8 (5.2%)	12 (15.4%)	ref	0.012
Mild/Moderate/Marked/Extreme	147 (94.8%)	66 (84.6%)	0.30 [0.12;0.77]	
Live in a city or by a busy area				0.440
Yes	123 (78.8%)	58 (74.4%)	ref	
No	33 (21.2%)	20 (25.6%)	1.29 [0.68;2.43]	
Live/work in a smoky atmosphere				0.266
No	115 (74.2%)	63 (80.8%)	ref	
Yes	40 (25.8%)	15 (19.2%)	0.68 [0.35;1.34]	
Exercise done				0.070
No	91 (58.7%)	36 (46.2%)	ref	
Yes	64 (41.3%)	42 (53.8%)	1.66 [0.96;2.87]	
Health status				0.580
Excellent + Very good + Good	57 (37.0%)	34 (43.6%)	ref	
Fair	86 (55.8%)	40 (51.9%)	0.78 [0.44;1.37]	
Poor	11 (7.1%)	4 (5.1%)	0.61 [0.18;2.07]	
Practice of vaginal douching				0.827
No	149 (95.5%)	74 (94.9%)	ref	
Yes	7 (4.5%)	4 (5.1%)	1.15 [0.33;4.06]	

Multivariate logistic regression analysis was then conducted to validate the pre-identified factors (from the univariate analysis) associated with DIE versus OMA. Women having any siblings (OR = 3.48, 95% CI: 1.59–7.64, *P* = 0.002), gastrointestinal symptoms during menstruation (OR = 2.09, 95% CI: 1.10–3.99, *P* = 0.025), or eating a greater number of fruit or vegetables (OR = 2.28, 95% CI: 1.04–4.97, *P* = 0.038) were more likely to be diagnosed with DIE. Meanwhile, eating organic food (OR = 0.18, 95% CI: 0.05–0.66, *P* = 0.024) and suffering from stress (OR = 0.28, 95% CI: 0.10–0.78, *P* = 0.015) were significant factors that were associated with the diagnosis of OMA (Table 4).

**Table 4:** Multivariate regression analysis of the factors associated with DIE vs OMA

Factors	OR (95% CI)	*P*
Having any siblings	3.48 (1.59, 7.64)	0.002
Gastrointestinal symptoms during menstruation	2.09 (1.10, 3.99)	0.025
No.of fruit/vegetables per day	2.28 (1.04, 4.97)	0.038
Stress level	0.28 (0.10, 0.78)	0.015
Eating organic food	0.18 (0.05, 0.66)	0.024
Hormonal treatment	2.02 (0.97, 4.20)	0.059
Infertility	1.34 (0.68, 2.64)	0.398
Previously surgical diagnosis	0.79 (0.34, 1.82)	0.573

## Discussion

The FEELING study was the first multinational, case-control observational study to investigate the potential factors associated with endometriosis development. The size and scope of the study enables a comprehensive assessment of the potential factors associated with DIE or OMA development across different countries. Here, we performed a subgroup analysis of the Chinese endometriosis participants to further investigate the potential factors associated with DIE versus OMA. Many factors were analyzed, with a special focus on the diagnosis, symptomatology and treatment practices in China. To the best of our knowledge, this is the first study that comparatively investigated the potential factors associated with DIE versus OMA in Chinese endometriosis population. Our results showed that women with any siblings, gastrointestinal symptoms during menstruation, and eating a greater number of fruit/vegetables were more likely to be diagnosed with DIE in the multivariate analysis. Meanwhile, eating organic food and suffering from stress were factors that were associated with the diagnosis of OMA.

DIE is generally known to be more symptomatic than other subtypes of the disease. The findings of our study showed that gastrointestinal symptoms during menstruation were a significant factor that is associated with the diagnosis of DIE versus OMA. Other pre-surgical complaints including infertility, pelvic pain, dysmenorrhea, deep dyspareunia and urinary symptoms were not found to be significantly associated with DIE when using OMA as control. These results were quite different from the study by Chapron et al., which indicated that in addition to gastrointestinal symptoms, complaints of painful symptoms (pelvic pain, dysmenorrhea, and deep dyspareunia) and lower urinary tract symptoms were also significantly more frequent in patients with DIE. Meanwhile, their study showed no significant difference in infertility rate among women with and without DIE [32]. However, an opposite result of increased infertility was observed in patients with DIE by Cornillie et al. study [33]. Additionally, the study by Cornillie et al. revealed that intensity of dysmenorrhea was closely related to the local invasion depth of endometriosis [34]. Women with DIE usually have sexual dysfunction and deep dyspareunia [26]. These conflicting results may be partially explained by ethnic differences and differences in the populations recruited.

The development of endometriosis has long been established to associate with various genetic and environmental factors, as well as lifestyle ones (diet habits and physical activity) [35, 36]. According to the FEELING study, clinical, environmental and lifestyle factors were potentially associated with OMA and DIE as a whole [30]. Dietary habits of the individuals may influence the physiological and pathological processes of endometriosis. However, studies on the disease in relation to diet have yielded equivocal results [37]. Increased consumptions of fruits and vegetables (preferably organic) were suggested to exert a protective effect on the development and progression of endometriosis [38, 39]. However, a significant increase in risk emerged for increased servings of fruit, as found by another study [40]. Subgroup analysis of DIE and OMA patients in our study indicated that a larger number of fruit/vegetables servings/day was independently associated with the diagnosis of DIE. Eating organic food is considered to exert a protective effect against the risk of developing endometriosis. The potential mechanisms may be associated with the normalization of estrogen production in women with endometriosis [41]. A complete change of diet was recommended for endometriosis patients, including eating mainly organic produce [42]. Our study showed that eating organic food was more likely to be associated with the diagnosis of OMA in the Chinese population. Furthermore, psychological stress seems to be high among women with endometriosis [43]. The severity of the disease, especially pain as the most relevant symptom, has been suggested to be the major reason for the elevated stress [44]. Women with DIE-related pain have been reported to have highest stress levels and a strong correlation between pain and stress was suggested [44]. However, women under stress in our population were significantly associated with the diagnosis of OMA. The potential reason is still elusive to us. The differences in pain perception among the countries or regions may play a role in this process. The lowest symptom perception was reported in Chinese women when compared with their French and Russia counterparts in FEELING study [8].

In addition to the factors mentioned above, other factors including age, BMI, marital status, smoking, blood type, educational level, prior pregnancy, co-morbidities, having been breastfed and a family history of endometriosis, were not found to be significantly associated with the diagnosis of DIE versus OMA. These results were partially consistent with the findings of some studies, which indicated no significant difference in smoking status and age and blood type distributions between women with OMA and DIE [45–48]. However, other studies also indicated lower BMI as a strong risk factor for DIE [49, 50]. A family history of endometriosis and use of oral contraceptives were reported to significantly associate with the incidence of DIE [31, 32]. However, no such association (family history of endometriosis, oral contraceptive and hormonal therapy use) was observed when OMA and DIE were compared in this study.

The wide-ranging clinical and environmental factors obtained in this study enable us to explore the potential factors associated with DIE versus OMA from multiple perspectives. Substantial differences were observed with respect to the diagnosis, symptomatology and management of DIE versus OMA, supporting the theory of complex and multifactorial origins of DIE and OMA.

The strengths of this study include the rigorous inclusion criteria (histologically confirmed OMA and/or DIE), and that comprehensive that were collected from a real-world setting. This study also has some limitations; the observational design means that cause and effect cannot be established between factors and outcomes. Furthermore, possible concomitant presence of the two subtypes of endometriosis in the DIE patients makes the conditions more complicated to study. For example, OMA has been reported to be associated with the severity of DIE [51], but the opposing results of no significant association between OMA presence and DIE severity have also been reported [52]. Taking into account the limitations of this study, further prospective studies are still needed.

## Conclusions

To sum up, the potential factors associated with DIE versus OMA were comparatively studied in the Chinese endometriosis participants from the FEELING study. Women eating a greater number of fruit/vegetables, having any siblings and gastrointestinal symptoms during menstruation were more likely to be diagnosed with DIE, while eating organic food and being under stress were major factors that potentially associated with the diagnosis of OMA. The results may help to better understand DIE versus OMA, and aid in earlier risk stratification and diagnosis of the patients.
